# Evaluation of postpartum anaemia screening to improve anaemia diagnosis and patient care: A prospective non-randomized before-and-after anaemia screening protocol implementation study

**DOI:** 10.1038/s41598-019-44334-9

**Published:** 2019-05-24

**Authors:** Enav Yefet, Abeer Suleiman, Gali Garmi, Aliza Hatokay, Zohar Nachum

**Affiliations:** 1 Department of Obstetrics and Gynecology, Emek Medical Center, Afula, Israel; 2 Rappaport Faculty of Medicine, Technion, Haifa, Israel

**Keywords:** Anaemia, Quality of life

## Abstract

We assessed the efficacy of a screening protocol for postpartum anaemia diagnosis and treatment in the maternity ward. A prospective non-randomized before-and-after anaemia screening protocol implementation study during two consecutive periods was conducted. Women who were scheduled for vaginal birth were tested for haemoglobin (Hb) before delivery. During the first period (June 29–October 10, 2015; N = 803) Hb was measured postpartum for women with anaemia-related symptoms, postpartum haemorrhage, or pre-delivery severe anaemia (Hb < 8 g/dL; “symptoms” group). During the second period (October 11, 2015–January 27, 2016; N = 755) Hb was also measured in all women with pre-delivery anaemia [i.e., Hb < 10.5 g/dL] (“screening” group). The primary outcomes were the rates of women with (1) postpartum anaemia diagnosis (Hb < 10 g/dL) and (2) administration of parenteral iron sucrose (indicated for postpartum Hb ≤ 9.5 g/dL). The detection rate of postpartum anaemia was higher in the screening group compared with the symptoms group (140 (19%) versus 100 (12%), OR_adjusted_ 2.2 95%CI [1.6–3.0], respectively). The iron sucrose treatment rate was also higher (110 (15%) versus 88 (11%), OR_adjusted_ 2.0 95%CI [1.4–2.7], respectively). A total of 122 women were diagnosed with moderate-severe anaemia in the screening group, 27 of whom (22%) were diagnosed solely due to the screening protocol. The results demonstrated that a routine screening of women with predelivery anaemia for postpartum anaemia led to increased anaemia diagnosis and consequently better medical care.

## Introduction

Anaemia during pregnancy is seen in 10–40% of the women. During pregnancy, anaemia is defined as haemoglobin (Hb) level < 10.5 g/dL. During the postpartum period, anaemia is defined as Hb level < 10 g/dL^1^. The primary aetiologies for postpartum anaemia are haemodilution, iron deficiency, anaemia during pregnancy, and ante-partum and postpartum haemorrhage. Anaemia is an important public health concern and if not diagnosed and managed appropriately might lead to short- and long-term complications such as dyspnoea, lethargy, dizziness, fatigue, decreased functional capacity, maternal infections, syncope, impaired quality of life, poor cognitive performance, emotional instability, low birth weight, preterm birth, increased risk for postpartum depression, impaired mother-child interactions, and increased mortality^1–10^.

Medical costs, including outpatient visits, emergency room visits, and inpatient admissions, for anaemic patients are as much as twice those for nonanemic patients with the same comorbid conditions^11^. Official guidelines recommend treating postpartum moderate to severe anaemia (defined as Hb level ≤ 9.5 g/dL) with intravenous iron supplements such as iron sucrose^12–14^, which was found to be superior to oral iron supplements in terms of more rapid rise in serum ferritin and Hb, and improved maternal fatigue scores in the postpartum period^3,13,15–17^. Accordingly, iron sucrose is routinely given in our department as a single dose of 500 mg for women with postpartum moderate-severe anaemia. Although the importance of treating postpartum anaemia is acknowledged, the literature does not provide clear recommendations how and when to screen for postpartum anemia^12,13,18^. Postpartum anaemia diagnosis can be challenging for a number of reasons. First, anaemia-related symptoms may be absent in women with ante-partum low Hb levels, or might be manifested only after patient discharge, when women experience more extensive physical activity. Second, anaemia-related symptoms can be attributed to weakness due to the process of childbirth and nursing, particularly if blood loss was not adequately assessed. Finally, clinical symptoms and signs can manifest late following substantial blood loss^19^ and in iron deficiency, which causes ineffective erythropoiesis, then reduced oxygen delivery to tissues, and only then do clinical symptoms and signs occur. The maternity ward stay is an important window of opportunity for anaemia diagnosis and medical intervention since the next point for medical assessment is only after 4–6 weeks and healthcare utilization can be absent in 10–40% of puerperal women depending on the specific area and population^20–22^.

In the present study we aimed to assess the efficacy of two different protocols for anaemia detection. In the first protocol, Hb was measured postpartum for women with anaemia-related symptoms, postpartum haemorrhage, or pre-delivery severe anaemia (Hb < 8 g/dL; “symptoms” group). In the second protocol, Hb was also measured in all women with pre-delivery anaemia [i.e., Hb < 10.5 g/dL] (“screening” group).

We hypothesized that in the screening group the rate of anaemia diagnosis will be higher, and consequently, more women will be treated with iron sucrose.

## Methods

### Design

A prospective non-randomized before-and-after anaemia screening protocol implementation study was conducted at Emek Medical Center, a university-affiliated hospital in Israel, from June 29, 2015 to January 27, 2016 (ClinicalTrials.gov Identifier: NCT02434653, date of registration: 28/04/2015). This study was authorized by the local review board of the Emek Medical Center (EMC 112-14) and was performed in accordance with relevant guidelines and regulations of our review board. Participants provided written informed consent.

Women who intended to or eventually delivered vaginally (spontaneous or by vacuum extraction) were tested for eligibility at the labour and delivery, maternal foetal medicine, or maternity wards. Inclusion criteria were women who delivered vaginally and age ≥ 18 years. Exclusion criteria were women under the age of 18 or who had known allergy to iron sucrose. We also excluded women who delivered by caesarean section because they have routine full blood count as part of their care, and women with pre-eclampsia with severe features, since Hb level is taken every 8 hours according to the departmental protocol. Recruitment took place in the delivery unit before delivery or after delivery at the maternity ward in two consecutive periods in which different intervention was performed for each (listed below in the following section) until the calculated sample size was obtained.

### Interventions

Hb level was obtained prior to or immediately after delivery from all the participants.

Women were divided into two groups.

1. “Symptoms” group – during the first period (June 29–October 10, 2015) additional tests for Hb levels postpartum were taken for women with anaemia-related symptoms, following postpartum haemorrhage, or for women with severe anaemia before delivery (defined as Hb < 8 g/dL).
2. “Screening” group – During the second period (October 11, 2015–January 27, 2016) Hb level was obtained prior to or immediately after delivery from all the participants. After delivery, Hb was measured for the same indications as the “symptoms” group and also for all women with peri-delivery anaemia (i.e., Hb < 10.5 g/dL) regardless of symptoms.

Hb tests were taken until Hb became stable – defined as less than 1 g/dL decrease between 2 tests at minimal interval of 8 hours. Hb tests were taken earlier according to the physician’s discretion in cases of substantial or active bleeding, severe symptoms consistent with haemorrhage or anaemia, or hemodynamic instability.

Treatment with iron supplements was performed according to the departmental protocol in both groups; women with Hb > 9.5 g/dL were treated with oral iron supplements (usually those that were taken during pregnancy), women with Hb ≤ 9.5 g/dL received in addition one dose of I.V. 500 mg iron sucrose. Women with Hb between 7 and 7.9 g/dL with anaemia-related symptoms or with Hb < 7 g/dL regardless of symptoms also received a transfusion of packed red blood cells.

### Study outcomes

The primary outcomes were (1) the rate of postpartum anaemia diagnosis (after birth), defined as Hb < 10 g/dL; and (2) the rate of women who received IV iron sucrose. Additional outcomes included the rate of patients who received a transfusion of packed red blood cells, the rate of postpartum haemorrhage (defined as decrease of 10% in haematocrit level from baseline), need for uterine revision, the mean number of blood tests that were performed during admission, mean and minimal Hb levels, and Hb level 6 weeks postpartum. We also collected data regarding iron sucrose adverse effects.

Six weeks postpartum we used a telephone questionnaire in which we asked women about the duration of breastfeeding, days of vaginal bleeding, and iron supplements consumption postpartum. They were also reminded to go to their community Ob/Gyn for routine check-up and to perform a complete blood count, according to our departmental protocol for postpartum recommendations.

### Statistical analysis

#### Sample size

A sample size of 748 women +15% drop outs for each group was calculated to find a 10% rate of postpartum anaemia in the symptoms group versus 15% in the screening group (5% one-sided alpha, power 90%) and an 8% rate of IV iron sucrose treatment vs. 12%, respectively (5% one-sided alpha, power 83%). Groups’ baseline characteristics and outcomes were compared using Student *t*-test (or Wilcoxon two sample test) for continuous variables and χ2 (or Fisher’s exact test) for categorical variables.

We controlled for background characteristics using multiple logistic regressions, and presented the adjusted odds ratios. We also performed a sub-analysis of the women who completed the study requirements with regard to Hb tests and iron sucrose treatment (data is not shown but is available upon request). Statistical analyses were carried out with SAS version 9.4 (SAS Institute, Cary, NC, USA). Significance was set at a *p* value less than 0.05.

The datasets generated and/or analysed during the current study are available from the corresponding author on reasonable request and subject to the directives of the institutional review board.

## Results

**Figure 1:**
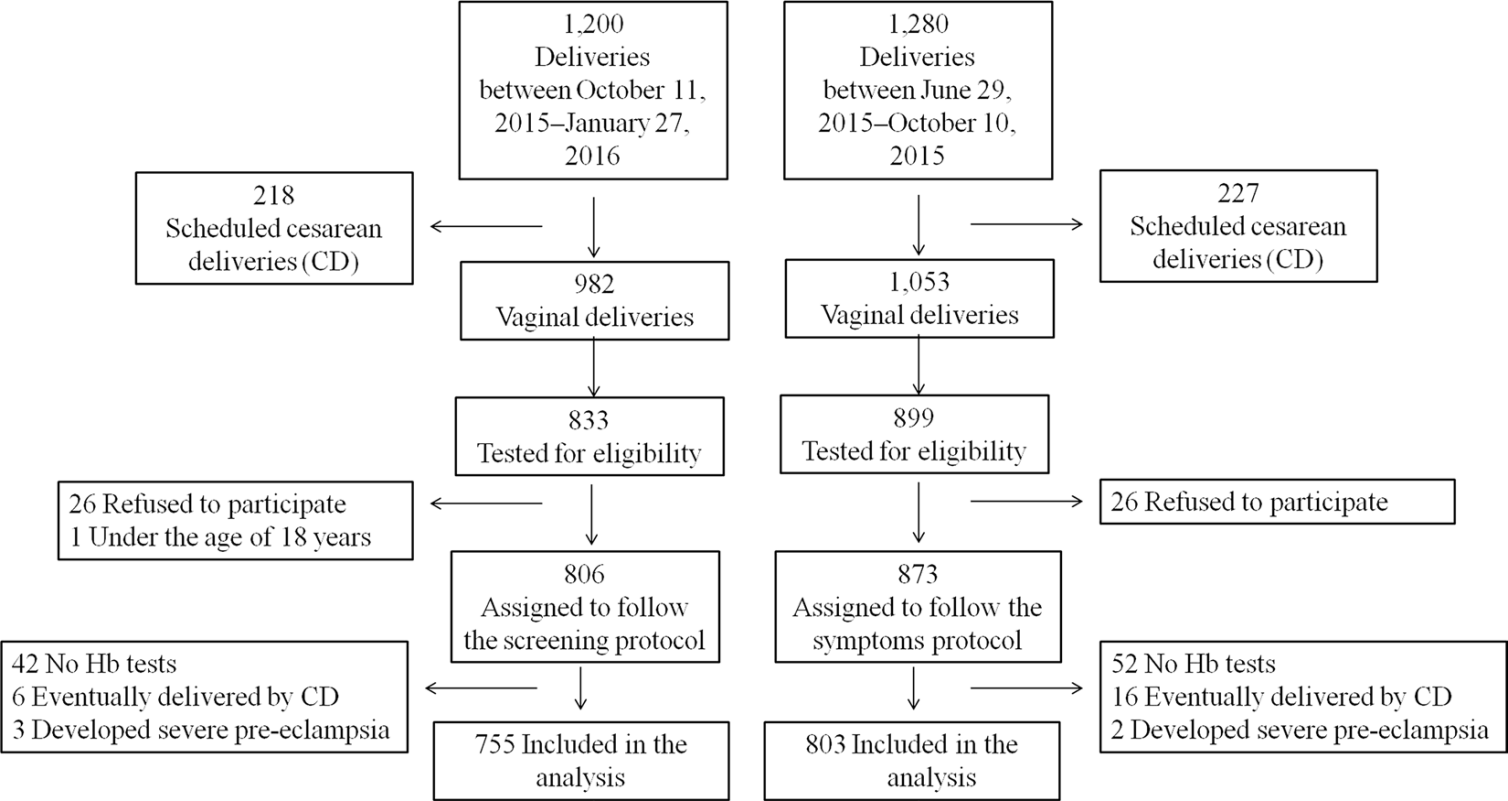
Patient flow chart.

Figure 1 shows the patient flow chart. Eight hundred and three women and 755 women were included in the final analysis of the symptoms and screening groups, respectively. Patients’ and neonates’ characteristics are summarized in Tables 1 and 2, respectively. The symptoms group included more women of Arab ethnicity or with beta thalassemia minor and anaemia prior to delivery.

**Table 1:** Patients’ demographic, pregnancy, and delivery characteristics and baseline quality of life survey scorings.

Group	Symptoms	Screening	p value
N	**803**	**755**	
Maternal age (years)	29 [25–33]	29 [25–33]	0.10
Number of delivery	2 [1–3]	2 [1–3]	0.18
Primiparity	271 (34%)	229 (30%)	0.15
Delivery week	39.3 [38.3–40.3]	39.4 [38.3–40.2]	0.52
**Ethnicity**			
Jewish	391 (49%)	414 (55%)	0.003
Arab	402 (50%)	321 (43%)	
Other	10 (1%)	20 (2%)	
Beta thalassemia minor	19 (2%)	7 (0.9%)	0.027
Smoking	3 (0.4%)	5 (0.7%)	0.50
**Place of residence**			
City >20,000 residents	403 (50%)	357 (47%)	0.25
Village ≤20,000 residents	400 (50%)	398 (53%)	
Pre-pregnancy BMI	23.0 [20.6–25.8]	23.1 [21.0–26.3]	0.10
Gestational diabetes mellitus	67 (8%)	46 (6%)	0.09
Pre-gestational diabetes mellitus	7 (0.9%)	5 (0.7%)	0.64
Chronic hypertension	8 (1%)	7 (0.9%)	0.89
Gestational hypertension	25 (3%)	26 (3%)	0.71
Epidural analgesia	303 (38%)	256 (34%)	0.12
Labour induction	298 (37%)	271 (36%)	0.62
Revision of uterine cavity and cervix (due to haemorrhage)	24 (3%)	35 (5%)	0.09
Manual removal of placenta	8 (1%)	11 (2%)	0.41
Vacuum extraction delivery	31 (4%)	29 (4%)	0.98
Prolonged second stage of delivery	18 (2%)	21 (3%)	0.50
Shoulder dystocia	6 (0.8%)	4 (0.5%)	0.75
Perineal tear ≥grade 3 (involving anal sphincter)	5 (0.6%)	4 (0.5%)	1
Episiotomy	77 (10%)	58 (8%)	0.18
HCT before delivery	35.1 [32.5–37.3]	34.9 [33.1–37.1]	0.30
Hb before delivery	11.8 [11–12.6]	12.1 [11.2–12.8]	0.0004
Hb < 10 g/dL before delivery	72 (9%)	37 (5%)	0.002
Hb ≤ 9.5 g/dL before delivery	35 (4%)	24 (3%)	0.22
Hb < 10.5 g/dL before delivery	114 (14%)	77 (10%)	0.016
MCH before delivery	28.4 [26.4–30.1]	29 [26.9–30.5]	<0.0001
MCHC before delivery	34.1 [32.8–35.1]	34.4 [33.4–35.2]	< 0.0001
Iron supplementation during pregnancy*	475 (92%)	508 (90%)	0.25

Values are presented as median [Interquartile range] or number (percent).

Pre-pregnancy BMI was missing for 10 participants.

*data was collected using questionnaire which was completed by 516 (64%) and 564 (74%) women in the symptoms and screening groups, respectively.

BMI, body mass index; Hb, haemoglobin; HCT, haematocrit; MCH, Mean Cell Haemoglobin; MCHC, Mean corpuscular haemoglobin concentration; Y, years.

**Table 2:** Neonates’ characteristics during pregnancy and delivery.

Group	Symptoms	Screening	*p* value
N	**841**	**765**	
Multiple gestation	22 (3%)	20 (3%)	0.91
Sex (males)	441 (54%)	419 (55%)	0.32
Birth weight (g)	3267 [3002–3552]	3256 [3000–3548]	0.80
Macrosomia (≥4000 g)	43 (5%)	37 (5%)	0.32
Apgar score at 1 minute	9 [9–10]	9 [9–10]	0.54
Apgar score at 5 minutes	10 [10-10]	10 [10-10]	0.46
Cord blood arterial pH	7.3 [7.2–7.31]	7.3 [7.21–7.31]	0.10

Values are presented as median [Interquartile range] or number (percent).

Apgar scores were not given to one neonate.

Arterial pH was not taken for 51 neonates due to technical reasons.

### Detection of postpartum anaemia and iron sucrose treatment

The study outcomes are summarized in Table 3. Adjusted odds ratio to ethnicity, beta thalassemia minor, anaemia prior to delivery, age, and BMI were calculated and are presented in Table 3. In the screening group, more women were diagnosed with postpartum anaemia (adjusted OR 2.2 95%CI [1.6–3.0]); moderate-severe anaemia (defined as Hb ≤ 9.5 g/dL) was diagnosed more in the screening group, including in women with baseline Hb > 9.5 g/dL, compared with the symptoms group (adjusted OR 1.8 95%CI [1.3–2.6]). In the screening group more women were documented with reduction in baseline Hb of at least 2 g/dL (adjusted OR 1.5 95%CI [1.1–2.1]).

**Table 3:** Signs, symptoms, and laboratory findings related to anaemia that were assessed after delivery.

Group	Symptoms	Screening	*p* value/OR_adjusted_ [95% CI]
N	**803**	**755**	
Peripartum anaemia (Hb < 10 g/dL) diagnosis*	137 (17%)	142 (19%)	1.6 [1.1–2.1]
Number of women diagnosed with postpartum anaemia after birth (Hb < 10 g/dL)	100 (12%)	140 (19%)	2.2 [1.6–3.0]
Detection of moderate-severe peripartum anaemia (Hb ≤ 9.5 g/dL)*	101 (13%)	121 (16%)	1.8 [1.3–2.5]
Number of women diagnosed with moderate-severe postpartum anaemia after birth (Hb ≤ 9.5 g/dL)	88 (11%)	119 (16%)	2.0 [1.5–2.8]
Number of women with baseline Hb > 9.5 g/dL who were diagnosed with Hb ≤ 9.5 postpartum	66 (8%)	97 (13%)	1.8 [1.3–2.6]
Reduction of postpartum Hb from Hb before delivery	1.3 [0.6–2.7]	1.5 [0.8–2.8]	0.40
Reduction of ≥2 g/dL	70 (9%)	96 (13%)	1.5 [1.1–2.1]
Reduction of ≥3 g/dL	40 (5%)	56 (7%)	1.5 [0.9–2.3]
Treatment with IV iron sucrose for postpartum anaemia	88 (11%)	110 (15%)	2.0 [1.4–2.7]
Total number of blood tests for Hb level (including follow-up tests)	1 [1-1]	1 [1-2]	0.001
Reasons for blood test for Hb**			
Clinical bleeding	48 (6%)	62 (8%)	0.09
Anaemia-related symptoms	60 (7.5%)	74 (7%)	0.10
Tachycardia	44 (5.5%)	52 (7%)	0.25
Low Hb^‡^	0 (0%)	66 (9%)	<0.0001
Other^§^	59 (7.4%)	25 (3.3%)	0.0004

Values are presented as median [Interquartile range] or number (percent).

OR (odds ratios) were adjusted to haemoglobin before delivery, beta thalassemia minor, maternal age, BMI, and ethnicity. They refer to the ratios between the screening and symptoms group for each outcome.

*Peripartum anaemia diagnosis was made according to haemoglobin levels before or after delivery.

**If a woman had more than one reason (e.g., clinical bleeding and tachycardia) she was counted in all the appropriate categories.

^‡^Low haemoglobin was considered <8 g/dL in the symptoms group and <10.5 g/dL in the screening group.

^§^Blood tests were obtained for other reasons, such as pre-eclampsia assessment or fever. Fewer were taken in the screening group since they were already available due to the screening protocol.

Hb, haemoglobin; HCT, haematocrit; OR, odds ratio.

In the screening group more women were treated with iron sucrose compared with the symptoms group (adjusted OR 2.0 95%CI [1.4–2.7]). Iron sucrose adverse effects were mild. Eleven women (5%) developed phlebitis in the injection site; three women (0.1%) reported paraesthesia; three women (0.1%) had pruritus, leg swelling, or rash; one woman (0.004%) reported dizziness and blurred vision; and one woman (0.004%) had shivering without fever. Except for phlebitis, the other adverse effects resolved after administration was discontinued.

Twenty-four women (1.5% of the study population) with Hb ≤ 9.5 g/dL were not treated with iron sucrose; 17 denied treatments, in 5 women the study protocol was not followed up, and in 2 women repeated Hb level was above 9.5 g/dL and therefore iron sucrose treatment was not indicated. Five women received partial treatment of iron sucrose; in 3 women treatment was discontinued due to adverse effect (shivering, rash, and phlebitis), one woman received 200 mg iron sucrose due to renal disease according to the nephrologist’s recommendation, and in another woman treatment was discontinued since she needed to be assessed for postpartum haemorrhage. In 76 women (4.5% of the study population) complete blood count was not taken even though the difference between the previous tests was at least 1 g/dL. The reasons for that were patient refusal to undergo additional blood exam, or in several cases where the tests were scheduled very late at night or early in the morning when the patients were asleep. In one patient from the screening group blood test was not taken even though baseline Hb was below 10.5 g/dL. Overall, 94% of the women completed the study requirements with regard to Hb tests and iron sucrose treatment. We performed a sub-analysis of those women (N = 1459). The results were similar to the results of the primary analysis (data not shown). The primary outcomes were also evaluated after excluding women with beta thalassemia minor. Similar results were obtained (data not shown).

The prevalence of blood transfusion (15 women (1.9%) versus 18 women (2.4%), *p* = 0.48), postpartum haemorrhage (30 women (4%) versus 41 women (5%), *p* = 0.10); residua more than 7 days after delivery (2 women (0.25%) versus 5 women (0.7%), *p* = 0.27) were infrequent and comparable between the symptoms and screening groups, respectively.

### The contribution of the screening protocol for anaemia diagnosis and treatment

In the screening group Hb level was obtained for 43 asymptomatic women with baseline Hb between 9.6 and 10.4 g/dL (in the symptoms group Hb measurement was not indicated for these women). Of them, 27 (51%) women had postpartum Hb ≤ 9.5 g/dL; thus, treatment with iron sucrose was indicated (the number needed to test is 1:2). A total of 122 women were diagnosed with moderate-severe anaemia in the screening group, 27 of whom (22%) were diagnosed solely due to the screening protocol.

### Long-term outcomes

Of the participants in this study, 89% and 93% of the symptoms and screening groups, respectively, were available for follow up after 6 weeks (Table 4). There was a slight increase in the duration of breastfeeding and slight decrease in the number of days of vaginal bleeding in favour of the screening group (Table 4). The rest of the parameters were not statistically different between the groups (Table 4).

**Table 4:** Questionnaire regarding bleeding, breastfeeding, iron consumption taken 6 weeks postpartum.

	N	Symptoms	N	Screening	*p* value/OR_adjusted_ [95% CI]*
Number of days of bleeding	714	21 [14–30]	703	20 [14–30]	<0.0001
Breastfeeding (days)	714	44 [30–53]	702	45 [30–55]	0.025
Currently breastfeeding	714	191 (27%)	703	193 (27%)	1.01 [0.8–1.4]
Full breastfeeding (days)		30 (26) [30]		29 (24) [30]	0.91
Breastfeeding ≥30 days	714	571 (80%)	702	552 (79%)	0.97 [0.7–1.3]
Iron supplementation during postpartum period	714	544 (76%)	702	545 (78%)	1.07 [0.8–1.4]

Values are presented as median [Interquartile range] or number (percent).

OR (odds ratios) were adjusted to haemoglobin before delivery, beta thalassemia minor, maternal age, BMI, and ethnicity. They refer to the ratios between the screening and symptoms group for each outcome.

After 6 weeks only 255 (32%) and 261 (35%) women in the symptoms and screening groups, respectively, performed a complete blood count. There was no difference between the groups (mean Hb 12.6 g/dL [SD 1.0] versus 12.5 g/dL [SD 0.97], respectively; *p* = 0.67).

## Discussion

In the present study we aimed to evaluate a postpartum anaemia detection program according to which all women with pre-delivery anaemia were screened for anaemia post-partum in the maternity ward. We found this protocol to be superior to our routine protocol of testing only symptomatic women, women with postpartum haemorrhage or severe pre-delivery anaemia with respect to postpartum anaemia detection and iron sucrose treatment. In the symptoms group more women were of Arab ethnicity, had beta thalassemia minor, and were diagnosed with anaemia prior to delivery. Since beta thalassemia minor is more prevalent in Arab women in our area those results make sense.

Although anaemia is associated with increased risk for significant morbidity and mortality^23,24^, there are no clear recommendations for postpartum anaemia testing^25^. Risk factors for postpartum anaemia were shown to be anaemia during the third trimester of pregnancy, postpartum haemorrhage, younger maternal age, and inadequate iron supplementation during the postpartum period^26,27^. While testing symptomatic women and following postpartum haemorrhage is insightful, to our knowledge a screening program to detect asymptomatic women for postpartum anaemia was not previously evaluated.

The screening protocol in the current study has led to a 22% increase in the diagnosis of moderate-severe anaemia. This result is significant since intravenous iron supplements are indicated to treat those women and are superior to oral iron supplements^3,13,16^. It should be mentioned that the difference between the number of blood tests between the two protocols was only 0.2 tests on average (22 cents per patient). In addition, 50% of the asymptomatic women with antepartum Hb between 9.6 and 10.4 g/dL had at least moderate-severe postpartum anaemia and received iron sucrose (number needed to treat 1:2). The small increase in the number of blood tests that has led to considerable improvement in anaemia diagnosis and treatment, suggests the screening protocol to be cost-effective.

The rates of blood transfusion or postpartum haemorrhage (i.e., decrease of 10% in haematocrit value from baseline) were not different between the groups. The data suggest that the reason was that Hb was already being tested due to symptomatic anaemia or clinical bleeding. Thus, the screening protocol increased the diagnosis rate of moderate to severe anaemia and the rate of iron sucrose treatment but not the diagnosis rate of severe anaemia, in which blood transfusion was indicated. In those cases anaemia-related symptoms are reliable. Those findings are consistent with the study of Dar *et al*. who demonstrated that a routine blood test as a single factor did not lead to blood transfusion^18^.

In this study, postpartum anaemia was treated empirically with iron supplements without testing for iron deficiency. Official guidelines recommend treating postpartum anaemia with iron sucrose according to Hb level alone without testing ferritin levels, since ferritin is an acute phase reactant and unreliable postpartum due to its physiologic increase in the first 6–12 weeks postpartum. The rationale for using iron sucrose treatment is to correct the acute blood loss during delivery and postpartum (haemorrhagic anaemia) and the high prevalence of iron deficiency anaemia during pregnancy^13,14^.

Therefore, in our opinion and based on the mentioned recommendations, it is justified to empirically treat those women with a single dose of 500 mg iron sucrose as long as this treatment is not contraindicated.

In this study women with thalassemia minor were included. The rate of thalassemia minor was very low in this study and accounted for 26/1558 women (1.7%). Women with thalassemia major were not included in this study. Since women with thalassemia minor suffer from mild anaemia and are not transfusion dependent, they usually do not suffer from iron overload. On the contrary, it was reported that about 30% of patients are iron deficient^28–31^. Since ferritin is physiologically increased postpartum, it is difficult to assess whether those patients are iron deficient or not. It is clear, however, that those patients experienced substantial blood loss due to the process of labour. To our knowledge there are no official guidelines how to treat those women with regard to iron supplements postpartum. In our opinion, in acute blood loss during and after delivery, a single administration of 500 mg iron sucrose in order to compensate for this blood loss is more beneficial than the risk of iron overload in thalassemia minor, which is uncommon.

The strengths of this study are its prospective design; the high recruitment rate, which decreased the selection bias; large sample size; the use of multiple aspects for evaluating the preferred protocol, which decreased the information bias, including multiple background characteristics; anaemia diagnosis; use of iron sucrose treatment; and addressing long-term effects. The limitations of this study are that it was not randomized, anaemia workup was not done in anaemic patients before delivery, and the low rate of women who performed complete blood count after 6 weeks. We chose to use a non-randomized design since the study involved a complex protocol that included a large number of medical and nursing personnel of the delivery unit and maternity ward. We assumed that by using a unified protocol for each period the risk for protocol violation could be minimized as indeed occurred. In addition, in both study periods similar number of deliveries occurred, anaemia is not likely to be affected by seasonal variations and the study outcomes were controlled for differences in patients’ characteristics. The fact that both primary outcomes were objective parameters with strict definitions also contributed to minimize the possible bias from non-randomized design. Another limitation was that the sample size was not sufficient to detect a difference in the rate of severe anaemia and need for blood transfusions. An important observation in this study is that only one third of the women performed a complete blood count 6 weeks postpartum, which is a routine recommendation in our department, even after we contacted them by phone. This low compliance of the mothers for medical care during the postpartum period stresses the importance of the maternity ward as an excellent window of opportunity for patient care. Future studies should consider screening for postpartum anaemia of asymptomatic women without pre-delivery anaemia, and also to perform anaemia workup in women with anaemia pre-delivery. Addressing anaemia during each stage of pregnancy and in the newborn should also be considered.

## Conclusion

Altogether, this study’s results suggest that routine postpartum screening of women with peri delivery Hb < 10.5 g/dL increased the detection rate of postpartum anaemia and particularly moderate-severe anaemia, leading to increased treatment with IV iron sucrose and improved patient care.
